# Affiliate stigma and associated factors among informal caregivers of people with mental illness in southwestern Uganda: A multi-center cross-sectional study

**DOI:** 10.1371/journal.pmen.0000132

**Published:** 2025-04-21

**Authors:** Alain Favina, Nicholas Hobe, Moses Muwanguzi, Gideon Munaru, Abel Rubega, Dan Lutasingwa, Joseph Kirabira, Godfrey Zari Rukundo, Scholastic Ashaba

**Affiliations:** 1 Department of Psychiatry, Mbarara University of Science and Technology, Mbarara, Uganda; 2 Department of Research, Health Development Initiative, Kigali, Rwanda; 3 Kisii Teaching and Referral Hospital, Kisii, Kenya; 4 Department of Psychiatry, Busitema University, Mbale, Uganda; 5 Department of Psychiatry and Behavioural Neurosciences, McMaster University, Hamilton, Ontario, Canada; Hamdard University - Islamabad Campus, PAKISTAN

## Abstract

The stigma surrounding mental illnesses is widespread and informal caregivers of patients with mental illness face stigma because of their relationship with the patients they care for. Despite the key role played by these informal caregivers in the management of people with mental illness, few studies have assessed affiliate stigma and its factors associated among this population. This study aimed to investigate the prevalence of affiliate stigma and associated factors among caregivers of patients with mental illness in southwestern Uganda. We used a cross-sectional study design and enrolled 385 caregivers. We assessed affiliate stigma, depression, and social support using the affiliate stigma scale, patient health questionnaire-9 and social support using the social provision scale respectively. We ran multivariable logistic regression models to assess for the factors associated with affiliate stigma among caregivers. The prevalences of affiliate stigma and depression were 65.97% and 25.2% respectively. Factors associated with affiliate stigma included caregiving for one year or longer (AOR: 1.89; 95% CI: 01.07–03.35; p = 0.03), having more than one patient to care for (AOR: 3.40; 95% CI: 01.39–08.36; p = 0.01), being the only caregiver to the patient (AOR: 2.60; 95% CI: 1.27–5.33; p = 0.01), being depressed (AOR: 75.76; 95% CI: 10.03–572.26; p < 0.001), and social support (AOR: 0.14; 95% CI: 0.06–0.29; p = 0.04). This prevalence of affiliate stigma among caregivers of patients with mental illness is high in southwestern Uganda and depression is a key predictor. Considering the important role played by informal caregivers, more studies are necessary to inform interventions to address affiliate stigma, depression, and overall mental health of caregivers.

## Introduction

Uganda is one of the six African countries with the largest burden of mental disorders where 1 in 5 of adults and children has ever suffered from mental illness [1]. Recent systematic reviews and prevalence surveys have demonstrated a high burden of depression, anxiety, psychosis and substance use disorders in Uganda [2–4]. Mental illness affects 24.2% of the adults and 22.9% of the children in the country, depressive disorders being the commonest severe condition with reported prevalence ranging between 14% and 30% [5–7]. A high burden of mental disorders in Uganda is linked to high levels of poverty and economic hardships, a high refugee population, HIV infection, emerging epidemics including Ebola and Covid-19, and civil wars [8–13]. Beliefs that associate medical disorders to shame and fear influence health seeking behaviour for mental illness and affect treatment outcomes [14]. It has been documented that in Uganda people with mental illness are guided by beliefs when seeking mental health services [15]. Most people believe that mental health problems are caused by evil spirits, witchcraft or ancestral spirits and hence delay in seeking mental health care while majority initially seek care from traditional healers [16,17]. Moreover, mental illness stigma is common and widespread and affects also informal caregivers of people with mental illness [18–20]. Stigma of mental illness is a major barrier to mental health care and it is associated with shame and rejection, and hence delay in seeking care [21]. Due to the stigma associated with mental illness, people with mental illness are at times abandoned by family which complicates their ability to access care [22] while some families hide their people with mental illness from the public and do not seek care to avoid disclosure and the stigma associated [17–23]. Mental illness stigma that affects informal caregivers through their association with people with mental illness under their care is referred to as affiliate stigma [24–26]. Informal caregivers of people with mental illness are family members, relatives, friends, or neighbors who provide ongoing assistance to the people with mental illness [27]. The concept of experiencing stigma because of an association with a person living with mental illness was first described by Goffman as courtesy stigma, stating that it involves public disapproval evoked as a consequence of associating with stigmatized persons [24–26]. The latter was later named affiliate stigma in subsequent studies [26]. Affiliate stigma consists of different elements including affective emotions such as a sense of dissatisfaction, cognitive factors such as feelings of helplessness, and behavioral factors such as avoidance or alienation due to stigma [28,29]. Affiliate stigma demonstrates the process by which caregivers of people with mental illness internalize the negative perception of themselves held by the public [30].

Affiliate stigma has been reported to affect more than half of the caregivers of people with mental illness [31–33]. In two studies conducted in Ethiopia, the prevalence of affiliate stigma ranges from 75% to 99.43% [18,34]. Additionally, two study conducted in USA and Singapore showed that affiliate stigma was prevalent at 66% and 94.5% respectively [35,36].

According to a recent systematic review [37], factors associated with affiliate stigma include the time spent caring for the patient, the disease attribution, stress, distress, fear of judgment from society, lack of support, and depression. Affiliate stigma as well as poor quality of life [36]. The association between depression and affiliate stigma has been linked to caregiver burden, involvement in care, and role, as well as the psychological distress mediated by the affiliate stigma [38,39]. Moreover, affiliate stigma causes feelings of low mood, guilt and shame, and helplessness owing to the responsibility of care, as caring for an individual with mental illness is lifelong due to the chronicity of mental illness and the stigma associated with it [19,26,40]. The relationship between the caregiver and the patient has also been reported to influence affiliate stigma experiences, with studies showing that parents of people with mental illnesses are more stigmatized than other family members (siblings and spouses) [36]. In addition, affiliate stigma is also associated with a reduction in the quality of life of caregivers of people with mental illness [36].

Informal caregivers collaborate with health care providers in the management of people with mental disorders by assisting patients with activities of daily living and providing instrumental support, such as transportation to the hospital, buying medicines, and ensuring that patients take prescribed medications [27,41,42]. A study on the mediating role of affiliate stigma on the relationship between caregiving burden and caregiver involvement in southwestern Uganda showed that informal caregivers experienced caregiving burden and that affiliated stigma impacted their caregiving roles and increased the burden of caregiving burden (39). The consequences of affiliate stigma therefore affect both the caregiver and the person with mental illness, with a negative impact on the overall treatment outcomes [43,44]. In addition to affecting the mental wellbeing of the caregivers, affiliate stigma disrupts family functioning and social life of those affected [19]. The emotional distress, low self-esteem, and caregiver burden resulting from affiliate stigma negatively impact the mental health outcomes of their family members with mental illness [45]. Family members are affected psychologically, physically, and due to the affiliate stigma, they have been reporting high prevalence of depression and psychological distress [33,46].

Additionally, with a high burden of care are more at risk of affiliate stigma [47]. Moreover, caregivers play a crucial role in the management of people with mental illness in Uganda [48,49]. However, despite the burden of affiliated stigma on the mental wellbeing and the caregiving burden of those affected including disruption of family functioning, information on affiliate stigma among people with mental illness in Uganda is limited.

To address this gap in literature, we aimed to determine the prevalence of affiliate stigma and associated factors among caregivers of people with mental illness.

## Methods

### Study design, setting and population

This cross-sectional study was conducted in southwestern Uganda among participants recruited from the mental health departments of three tertiary hospitals and one private hospital. These hospitals included Mbarara Regional Referral Hospital (MRRH), Kabale Regional Referral Hospital (KRRH), Masaka Regional Referral Hospital, and Kampala International University Teaching Hospital (KIU-TH) (a private hospital). The four tertiary hospitals are the only hospitals with psychiatry units serving people with mental illness in southwestern Uganda. In these hospitals, patients attend regular reviews for follow-up in the outpatient department (OPD) and nearly all patients are accompanied by a caregiver who assists in presenting the patient’s concerns.

### Study population and sample size estimation

We enrolled 385 informal primary caregivers consecutively until the predetermined sample size was reached. The sample size was computed using a population size large than 1 million, a hypothesized frequency of outcome factor in the population of 50%, a confidence level of 95% and a margin error of 5%. We included participants who were aged 18 years and older, were providing care for a patient with a mental illness for at least 3 months and provided consent to participate in the study. We considered a period of three months as sufficient for the caregiver to have had enough experience in that role and able to answer the research questions.

### Enrollment procedure

After the regular clinic review of their patients, caregivers were approached. Participants were informed about the study purpose and proposed to participate voluntarily in the study. Those who chose to participate were required to provide written informed consent. Trained research assistants then administered a questionnaire in Runyankore or English, depending on the participants’ preference in a private room. The enrollment procedure took place from 7^th^ February to 28^th^ June 2023.

### Ethical considerations

The present study was conducted in accordance with the Declaration of Helsinki 2013 [50]. The study was approved by the Research Ethics Committee of the Mbarara University of Science and Technology (MUST-2022–609) and the hospital administrators provided clearance. All participants provided written informed consent before enrollment. The explanation of the goal of the study, and the consent process was witnessed and documented by the research assistants.

### Study variables

Outcome variable in this study was affiliate stigma measured using the affiliate stigma scale.

Independent variables assessed included socio-demographic information such as age (in completed years), sex (male, female), marital status, highest level of education, the relationship with the patient, duration of caregiving, number of days of caregiving in a week, number of months on caregiving, the diagnosis the patient is being managed for as per medical record, number of patients one is caring for, number of caregivers for the patient. In addition, we collected information on social support using social provision scale (SPS) and depression using the patient health questionnaire tool (PHQ-9).

### Study measures

#### Affiliate stigma scale.

Affiliate stigma was assessed using the affiliate stigma scale [26]. The scale has 22 items rated on a 4-point Likert scale with a score of 31 and above representing a positive screen for affiliate stigma. The scale has demonstrated good psychometrics properties in previous studies [28,51] and in this study the scale had a Cronbach’s alpha of 0.92.

#### Social support.

The social provision scale was used to determine social support among the study participants. The social provision scale (SPS) is a 24-items scale [52]. The SPS scale is rated on a four-point Likert scale, with 1 representing “never” and 4 representing “always.” The scale was measured as a continuous variable, with a higher score indicating stronger social relationships and a low score indicating loneliness [53]. In this study, the scale had a Cronbach’s alpha of 0.91.

#### Depression.

Depression was measured using the PHQ-9, a 9-item depression screening tool. Each of the 9 items is scored on a 4-point Likert scale ranging from 0 (“not at all”) to 3 (“nearly every day”). The PHQ-9 total score ranges from 0 to 27 with a score of 10 and above representing a positive screen for depression (Kroenke et al., 2001). The PHQ-9 has been validated in African settings including Uganda with good psychometric properties [54,55]. In the present study, the Cronbach alpha was 0.89.

#### Data analysis.

The data was analyzed using STATA Version 17 [56]. Participants’ demographics were summarized using descriptive statistics, where normality of continuous variables was tested basing on the Gaussian assumption using the Shapiro-Wilks test, and the histograms and was normally distributed. Thus, age and social support were presented with means and standard deviations (SD). The categorical variables were presented as frequencies and percentages. To compare the distribution between females and males, we used the Student t-test for continuous variables, and Chi square or Fisher’s exact test for categorical variables. Bivariate and multivariable logistic regression analyses were performed to assess for the factors associated with affiliate stigma. We considered a 95% confidence interval and a margin of error of 5%.

## Results

### Sociodemographic characteristics

A total of 385 caregivers were enrolled. The mean age of the participants was 45.63 (SD 14.13) years, and the majority of caregivers were women (237/385, 61.56%). The majority (250/385, 64.94%) of caregivers were unemployed and 43.38% (167/385) of the participants were caring for patients with bipolar disorder. About a quarter (25.2%) of caregivers had depression. More than half of caregivers (254/385, 65.97%) had affiliate stigma (Table 1).

**Table 1:** Characteristics of study participants (n = 385).

	Total	Female	n = 237 (61.56%)	Male	n = 148 (38.44%)	X2 (P value)	
**Age *mean (SD)***	45.6	−14.1	45.7	−14	45.5	−14.4	(0.88)
**Marital Status**							
Divorced/widowed	58	15.10%	53	22.40%	5	3.40%	**25.79 (<0.001)**
Married/cohabiting	281	73.00%	157	66.20%	24	83.80%	
Single	46	11.90%	27	11.40%	19	12.80%	
**Address**							
Urban	83	21.60%	54	22.80%	29	19.60%	0.55 (0.46)
Rural	302	78.40%	183	77.20%	119	80.40%	
**Level of education**							
None	37	9.60%	32	13.50%	5	3.40%	**23.41 (<0.001)**
Primary	182	47.30%	123	51.90%	59	39.90%	
Secondary	109	28.30%	56	23.60%	53	35.80%	
Tertiary	57	14.80%	26	11.00%	31	20.90%	
**Employment status**							
Employed	135	35.10%	57	24.10%	78	52.70%	**32.85 (<0.001)**
Unemployed	250	64.90%	180	75.90%	70	47.30%	
**Relationship with the patient**							
Child (Daughter or son)	18	4.70%	6	2.50%	12	8.10%	**26.52 (<0.001)**
Spouse	48	12.50%	28	11.80%	20	13.50%	
Sibling (Sister or brother)	113	29.40%	59	24.90%	54	36.50%	
Parent (Father or mother)	178	46.20%	132	55.70%	46	31.10%	
Other (Friend, Grandparents and in law, uncle, aunt)	28	7.30%	12	5.10%	16	10.80%	
**Years of caregiving**							
Less than a year	138	35.80%	85	35.90%	53	35.80%	0.0001 (0.99)
A year and above	247	64.20%	152	64.10%	95	64.20%	
**Number of caregiving day/week**							
1–4	84	21.8%	36	15.20%	48	32.40%	**15.88 (<0.001)**
5–7	301	78.20%	201	84.80%	100	67.60%	
**Disorder of the patient being caring of**							
Major depressive disorder	35	9.10%	21	8.90%	14	9.50%	5.02 (0.29)
Psychiatric disorder secondary to medical condition	34	8.80%	24	10.10%	10	6.80%	
Substances related disorder	55	14.30%	33	13.90%	22	14.90%	
Schizophrenia and other related primary psychosis	94	24.40%	50	21.10%	44	29.70%	
Bipolar affective disorder	167	43.40%	109	46.00%	58	39.20%	
**Number of patients caring of**							
More than one	48	12.50%	34	14.30%	14	9.50%	1.99 (0.16)
One	337	87.50%	203	85.70%	134	90.50%	
**Being the only caregiver**							
Yes	104	27.00%	62	26.20%	42	28.40%	0.23 (0.63)
No	281	73.00%	175	73.80%	106	71.60%	
**Social support *mean(SD)***	3.1	−0.3	3.1	−0.3	3.1	−0.3	−0.73
**Depression**							
No	288	74.80%	175	73.80%	113	76.40%	0.30 (0.58)
Yes	97	25.20%	62	26.20%	35	23.60%	
**Affiliate stigma**							
No	131	34.00%	79	33.30%	52	35.10%	0.13 (0.72)
Yes	254	66.00%	158	66.70%	96	64.90%	

#### Factors associated with affiliate stigma among informal caregivers of patients with mental illness.

At multivariable regression analysis, the factors statistically significantly associated with affiliate stigma were caregiving for one year or longer (AOR: 1.89; 95% CI: 1.07–3.35; p = 0.03) having more than one patient to care for (AOR: 3.40; 95% CI: 1.39–8.36; p = 0.01), being the only caregiver to the patient with mental illness (AOR: 2.60; 95% CI: 1.27–5.33; p = 0.01), social support (OR: 0.14; 95% CI: 0.06–0.29; p = 0.04) and having depression (AOR: 75.76; 95% CI: 10.03–572.26; p < 0.001) (Table 2)

**Table 2:** Associated factors to affiliate stigma using bivariate and multivariate analysis.

	Bivariate analysis	Multivariate analysis				
Crude OR	95%CI	P-value	Adjusted OR	95%CI	P-value	
**Age**	1.02	1.01–1.04	**0.01**	1.01	0.98–1.03	0.57
**Gender**						
Female	Ref			Ref		
Male	0.92	0.60–1.4	0.72	0.92	0.51–1.68	0.79
**Marital Status**						
Divorced/widowed	Ref			Ref		
Married/cohabiting	0.84	0.46–1.54	0.58	1.43	0.63-3.24	0.39
Single	0.93	0.41-2.13	0.86	2.62	0.77-8.92	0.12
**Address**						
Urban				Ref		
Rural	1.47	0.89–2.42	0.13	1.81	0.92–3.57	0.09
**Level of education**						
None	Ref			Ref		
Primary	0.74	0.33–1.68	0.47	1.17	0.42–3.24	0.77
Secondary	0.47	0.20–1.10	0.08	0.9	0.29–2.84	0.86
Tertiary	0.48	0.19–1.19	0.11	0.79	0.22–2.80	0.71
**Employment status**						
Employed	Ref			Ref		
Unemployed	1.50	0.97–2.32	0.07	0.7	0.37–1.33	0.27
**Relationship with the patient**						
Child (Daughter or son)	Ref			Ref		
Spouse	0.91	0.29–2.86	0.87	0.92	0.21–4.06	0.91
Sibling (Sister or brother)	0.65	0.23–1.86	0.43	0.92	0.25–3.47	0.91
Parent (Father or mother)	1.15	0.41–3.22	0.79	1.04	0.26–4.15	0.95
Other (Friend, Grandparents and in law, uncle, aunt)	2.30	0.58–9.11	0.24	1.84	0.34–9.85	0.48
**Years on caregiving**						
Less than a year	Ref			Ref		
A year and more	2.21	1.43–3.42	**<0.001**	1.89	1.07–3.35	**0.03**
**Number of caregiving hours day/week**	1.09	0.96–1.22	0.18			
1–4 Days	Ref			Ref		
5–7 Days	1.34	0.81–2.21	0.251	0.94	0.48–1.84	0.86
**Diagnosis of the patient**						
Major depressive disorder	Ref			Ref		
Psychiatric disorder secondary to medical condition	2.89	0.99–8.41	0.05	2.44	0.71–8.46	0.16
Substances related disorder	2.20	0.89–5.42	0.08	1.15	0.38–3.46	0.81
Schizophrenia and other related primary psychosis	2.07	0.92–4.6	0.08	1.32	0.49–3.54	0.58
Bipolar affective disorder	1.04	0.50–2.17	0.918	0.55	0.22–1.41	0.21
**Number of patients caring for**						
One	Ref			Ref		
More than one	2.87	1.30–6.34	**0.01**	3.4	1.39–8.36	**0.01**
**Being the only caregiver**						
No	Ref			Ref		
Yes	2.96	1.71–5.15	**<0.001**	2.6	1.27–5.33	**0.01**
**Social support**	0.14	0.06–0.29	**<0.001**	0.37	0.14–1.00	**0.04**
**Depression**						
Not Depressed	Ref			Ref		
Depressed	78.99	10.86–574.23	**<0.001**	75.76	10.03–572.26	**<0.001**

## Discussion

In this cross-sectional study, we determined the prevalence of affiliate stigma and its associated factors among caregivers of people with mental illness in southwestern Uganda. The prevalence of affiliate stigma was high (65.97%) and the associated factors included depression, caregiving for one year or longer, being the only caregiver for the patient, having more than one patient to care for, and social support.

The high prevalence of affiliate stigma in our study compares with what was reported in a study conducted in the USA, where 66% of caregivers involved in caring for people with bipolar disorder reported experiences of affiliate stigma [35]. However, the prevalence in our study was lower than that reported in the two studies conducted in Ethiopia (75% and 99.43%) and another one in Singapore (94.5%) [18,34,36]. The majority of the caregivers in our study were caring for patients with bipolar disorder. Schizophrenia has been associated with higher levels of stigma because it is a serious and chronic mental illness that requires intense caregiver involvement [57]. Additionally, people with schizophrenia are perceived by the public to be more dangerous and hence these patients and their caregivers are faced with public stigma and discrimination compared with patients with other conditions like bipolar disorder or depression because they easily internalize public perceptions [19]. Moreover, majority of the caregivers in our study were women who are accustomed to caregiving roles in this setting [58,59]. Having the majority of caregivers being women could have reduced the prevalence of affiliate stigma since women are more resilient and accommodative in their caregiving roles [60] hence the lower prevalence compared that documented in Ethiopia and Singapore [18,34,36]. However, in a previous study it was that documented that female caregivers are at a high risk of affiliate stigma mainly because women are at a high risk of psychological morbidity [61] The lower prevalence of affiliate stigma in our study compared to the studies in Ethiopia may also be due to the fact that Uganda has been making efforts over the last year to spread awareness about mental illness stigma through community-led mental health interventions that have contributed to the reduction of mental illness stigma [62–66]. These interventions have equipped people with knowledge about mental illness, bearing in mind that lack of knowledge about mental health is one of the factors associated with high levels of stigma [37,67]. On the other hand, the prevalence was higher compared to other more recent studies conducted in Ethiopia (54.9% and 41.5%) [68,69]. The differences are likely due to cultural differences and differing beliefs about the causes of mental illness [70]. In Uganda, people with mental illness are often rejected and discriminated making their family members fearing to mix with other people in the community [49]. Moreover, other studies have indicated that affiliate stigma is common among women caregivers who are older [34] and the mean age of our participants was 45.6 years and majority were women which may explain a higher prevalence of affiliate stigma among our study participants.

In our study, having depression was associated with an increased risk of having affiliate stigma. An association between affiliate stigma and depression among caregivers of people with mental illness has been reported in previous studies [20,33,46,71–73] and this has been linked to the burden of care, which tends to be lifelong and comes with emotional drain, public scrutiny, and discrimination [19,26,38–40]. In addition, a previous study revealed that affiliate stigma partially mediates the association between caregivers’ depression and self-esteem [46]. Overall, caregivers of patients with mental illness often have a high burden of mental health difficulties compared to the general population [74] due to the stress experienced in the caregiving process as their own needs are not given attention [29,75]. This finding highlights the need to pay attention to the caregivers of people with mental illness, first by assessing for affiliate stigma and the associated mental health problems and addressing them. This will help caregivers in their caregiving role and reduce the burden of caregiving.

Being the only caregiver for the patient with mental illness was associated with an increased risk of affiliate stigma. This result is similar to the one found by Minichil and colleagues [20]. This echoes findings of a previous study by Kaggwa and colleagues in southwestern Uganda in which affiliate stigma was found to serve as a full mediator between the caregiver’s roles and involvement in care [39]. Being the only caregiver, increases the burden of care in terms resources invested including time, physical presence, social, and financial resources [76]. Caring for a patient with mental illness requires commitment and is not only multidimensional but equally challenging [49]. However, being the only person caring for the patient may make the caregiver-patient relationship strong with a feeling of being “connected” and this can promote patient recovery and functionality [77]. On the contrary, having more family members involved in the care of the person with mental illness provides social support which influences positivity, may lower caregiving stress and affiliate stigma and improve wellbeing of the caregivers [26].

Our current findings also showed social support was protective against affiliate stigma. Similar findings were documented in studies conducted in Ethiopia, where caregivers who had poor social support faced more stigma compared to people with good social support [20,68,78]. Having social support increases the resilience to stigma and reduces the physiological and psychological effects of stress, with an increase in the overall quality of life of the caregivers [79,80]. Social support means acceptance in society despite the challenges, and it is a source of comfort that reduces experiences of stigma [81].

In our study, we also found that caring for more than one patient increases the risk of having affiliate stigma. Previous studies have demonstrated that having many people to care for increases the burden of caregiving through the higher number of hours spent caring, high psychological distress, social isolation, financial stress, and lack of choice in being a caregiver [26,37,39,61,82]. This makes the caregiver stay under constant stress, affecting the caregiver’s health and wellbeing [83]. In addition, the person caring for many patients may be affected by the cultural beliefs in that people in communities believe that many people with mental illness in a single household is due to curses and misfortunes [84,85] which may cause social isolation and lack of social support from the community and hence increased stress [86,87].

Caring for a patient for a year or longer was also associated with an increased risk of having affiliate stigma. This finding reflects findings of previous studies where care duration and care time per day were associated with stigma among caregivers [26,70,78]. The higher number of hours spent caring increases the burden of caregiving, and as a consequence, caregivers may abandon other personal activities leading social isolation [37]. However, the findings differ from findings of a study in India which showed short being in the caregiving role for a short duration and spending more time on caregiving [61]. The relationship between the short duration of caregiving and affiliate stigma among caregivers has been linked to the fact that caregivers get into the caregiving role expecting the patient’s condition to improve faster, and when this doesn’t happen, the caregivers experience stress, doubt their ability to take on a caregiving role, and thus psychological distress and affiliate stigma [61].

Unlike earlier studies [19,29], we did not find an association between the patient’s type of mental illness and affiliate stigma among caregivers. This is likely because the majority of the caregivers in our study were taking care of patients with bipolar disorder which less stigmatized than schizophrenia [19]. Schizophrenia is the most stigmatized of the mental disorders, and individuals with this condition endure the serious consequences that come with it, especially stigma and this stigma also spills over to the caregiver [70,88]. Caregivers of patients with schizophrenia experience affiliate stigma due to the internalization of the stereotypes, labeling, and discrimination from the public, hence affiliate stigma [89,90]. Because schizophrenia is the most chronic mental illness and the burden of care worsens with severe the symptoms of illness which leads affiliate stigma especially when caregivers are faced with uncertainty about the future in the caring process [91].

We did not find an association between the relationship between the patients and the caregiver and affiliate stigma. A previous study a documented an association between caregiver relationship with the patient and affiliate stigma noting that caregivers who are parents to the person with mental illness are more at risk of affiliate stigma compared to other caregivers [19]. This is reported to be due to cultural beliefs related to the cause of the mental illness where by parents are anticipated to be responsible for the wellbeing of their children and the presence of mental illness in their child is viewed as failure on their part [92]. As a result caregivers who are parents to the patient with mental illness tend to blame themselves for having not done enough to protect their child resulting in negative emotions, social isolation and hence affiliate stigma [93]. Our findings are likely different due to the fact that majority of the caregivers were women and previous research in the same setting showed that women caregivers had low levels of caregiver burden compared to men [39]. Other studies in Uganda have also shown that women in this setting are the key caregivers proving day to day caregiving as a result of their upbringing and nurturing through they have gained good coping skills than men [58,94].

## Limitations

When interpreting our findings, the following limitations should be considered. This was a cross-sectional study, and causality could not be determined. Further studies using other study designs, such as cohort design studies, need to be conducted to determine the causal relationship between affiliate stigma and other associated factors. In addition, participants in this study were enrolled from the outpatient clinics and the patients under their care were in remission. Further studies involving caregivers of both patients in remission and with active symptoms may provide further understanding of affiliate stigma among caregivers. Additionally, longitudinal studies to track changes in stigma over time or intervention studies aimed at reducing stigma could provide valuable insights for practitioners and policymakers.

## Conclusion

The current finding showed a high prevalence of affiliate stigma among caregivers of people with mental illness in southwestern Uganda and depression is a key predictor. Health care providers should consider screening for stigma and depression among caregivers of patients with mental illness. There is also a need to design interventions that promote social support for caregivers of patients with mental illness.
